# A Study on Egg Production and Quality According to the Age of Four Italian Chicken Dual-Purpose Purebred Hens Reared Outdoors

**DOI:** 10.3390/ani13193064

**Published:** 2023-09-29

**Authors:** Chiara Rizzi

**Affiliations:** Department of Agronomy, Food, Natural resources, Animals and Environment (DAFNAE), University of Padova, Viale dell’Università, 16, 35020 Legnaro, Italy; chiara.rizzi@unipd.it

**Keywords:** hen, local breed, age, photoperiod, egg production, egg quality

## Abstract

**Simple Summary:**

The onset of egg laying for hens is affected by genotype, nutritional body status and environmental conditions. Local breeds (white eggshell breeds—Padovana and Polverara; tinted eggshell breeds—Pepoi, Ermellinata di Rovigo, Robusta Maculata and Robusta Lionata) from the Veneto region, in Northern Italy, were reared outdoors from spring (6 weeks of age; 6 WA) to autumn and winter. The onset of laying varied according to the breed (22–28 WA): only the tinted eggshell breeds started laying at the end of summer/beginning of autumn, in the presence of a decreasing natural photoperiod, and the egg production lasted for some weeks. At 38 WA, artificial light was gradually added to the photoperiod to obtain 14L:10D, and at 42 WA, all six breeds were laying. For hens reared outdoors, interactions among environment, body energy and nutritional requirements should be considered. The data on the productive yield and egg quality of the local breed hens are useful for the management of the birds and for an adequate supply of eggs, both for market and for brooding throughout the year.

**Abstract:**

The month of hatching and the rearing management, especially temperature and photoperiod, are important factors for pullets and hens reared outdoors. The yield performance and egg quality of dual-purpose chicken breeds from the Veneto region (Italy), Pepoi (PP), Ermellinata di Rovigo (ER), Robusta Maculata (RM) and Robusta Lionata (RL), with different adult body weights (ABW, kg, PP = 1.3; ER = 2.3, RM and RL = 3.1), were studied, using a factorial model (4 × 2), considering breed and age (26–33 weeks, first age, summer–autumn, under decreasing natural photoperiod—on average, 12L:12D, and 42–53 weeks, second age, winter, under implemented photoperiod-14L:10D) as the main effects and interaction. The chicks hatched in spring, and they started laying at the end of summer/beginning of autumn. Significant (*p* < 0.05) results were shown for many traits. ER showed higher hen–day egg production than that of PP, and RM and RL were the lowest; ER, RM and RL showed medium-size eggs and PP showed small-size eggs. RM produced the most spherical eggs and ER the most ovoid, and they showed the highest and the lowest eggshell thickness, respectively. RM showed the highest yolk to albumen ratio, and RL showed the lowest. The age increased the laying rate and the egg weight in all the groups. At 26–33 weeks, ER and PP showed higher hen–day egg production (on average 24%) than RM and RL (on average, less than 10%). The onset of laying (at least 10% laying rate) was shown, at different ages, according to the % ABW the breeds had reached: PP was the first, followed by ER, then RM, and RL was the last. At 42–53 weeks, the hen–day egg production ranged, on average, from 38 to 52%, according to the breeds; orthogonal contrasts on two-weekly data showed, at first age, increasing linear (ER) and quadratic (other groups) trends, and at second age, positive linear (ER, RM) and cubic (PP, RL) trends. Age (32 vs. 53 weeks) affected almost all the eggshell traits in PP and ER, whereas in RL, and especially RM, fewer traits changed. The age increased the yolk to albumen ratio (unchanged in PP). These results may be useful for the effective management of local purebred chickens, with the purpose to ensure the wellbeing of the hens and for supplying eggs of different quality throughout the year.

## 1. Introduction

Throughout the last few decades, interest in dual-purpose chicken breeds has been increasing, both for biodiversity and ethical concerns. Given the progress in genetic improvements for yield performance in hybrid hens carried out by a few breeding companies all over the world [1], biodiversity is currently one of the key principles of sustainability for food and agriculture [2]. The ethical impact on animal welfare is a further important concern, which considers not only the rearing conditions of the birds but also their management. The males of egg layer hybrids are killed after hatching, as they do not perform the standard growth and slaughtering quality requested for the market [3]. Furthermore, hybrid broilers show many physical problems, which do not allow them to have satisfactory well-being conditions, and free-range rearing systems are increasing as they allow chickens to better show their natural behaviour [3,4]. In Italy, the Veneto region has a long tradition of poultry breeding, a few chicken breeds have existed for many centuries, and in the middle of the last century, some dual-purpose purebreds were created using white- and brown-eggshell breeds [5]. The first character that distinguishes the breeds, according to their origin, is the colour of the eggshell, as chicken genotypes have a different phylogenetic origin, having evolved separately from the wild *Gallus* genus after domestication, and many body characteristics differ between them. Following the classification suggested by Ghigi, in 1905, the domestic chicken breeds can be divided into three groups, according to their morphological, biological and functional characteristics [6]. There are breeds, which are like wild *Gallus* genus, showing early growth and sexual maturity and a high production of eggs with white eggshells. On the contrary, there are breeds highly different from wild *Gallus*, showing delayed growth and sexual maturity, low egg production with brown eggshells, and, in the third group, there are intermediate breeds, known as dual-purpose breeds, which show intermediate traits, with more similarity to the breeds belonging to the first or second group [6]. In fact, it is known that the breeds producing eggs with white eggshells have body growth, a metabolic profile and physiology more suited to higher egg production than brown eggshell breeds. This physiological condition, well exhibited by white eggshell hybrids [7,8], demonstrates that the nutrient intake is used for most of the skeletal and muscle growth until the onset of laying and then used for egg production, avoiding competition for the ingested nutrients between body development and egg formation. More recently, studies, based on SNP array, genotyped many chicken breeds and hybrids across the globe to test the genetic diversity between and within the populations for the effective management of chicken genetic resources [9,10]. Differences in genetic diversity within the population were shown between the highly selected commercial layer lines and many African, South American and some local Asian and European breeds [9], as well as some Italian breeds [10].

Lately, interest in local products has been increasing [11]. Egg production from local purebreds is still a niche form of production, which shows variability in the management of birds throughout their growth and laying cycle and in the egg supply for marketing. Nonetheless, for dual-purpose breeds, the availability of chicks for meat production is a limitation for the management of the farm [12]. For birds reared outdoors, variations in the onset of laying and on the hen–day egg production may occur according to the month of hatching [13], as well as the age, which varies among genotypes [14]. Furthermore, the older a pullet when the first egg is laid, the larger the eggs during the laying period [15]. Thus, the delay in the onset of egg production and the age association, generally, become important economic issues. In the presence of extreme environmental temperatures, as occur in countries with hot summers and cold winters, the season affects performance, involving relevant changes in egg size and feed consumption, with minimal effects on egg production [14]. Knowledge on the environmental factors and age at photostimulation, which affect the onset of laying, egg production and egg traits, mainly considers hybrid hens [16,17], whereas the data on purebreds are scant [18]. Furthermore, it is known that the yield performance of hybrids is higher than that of purebreds [5,8]; thus, it appears opportune to investigate the egg production and egg quality of different local breeds, undergoing conservation plans for their importance on poultry biodiversity [19], according to the month of hatching and rearing season, with the purpose to achieve knowledge for the best management programme for the birds and their products and for prospective novel selection schemes.

The aim of this work is to study the effect of breed and age on the egg production and the egg external and internal quality in four Italian dual-purpose breeds [19], at the beginning and some months later than first weeks laying activity that occurred, respectively, at the end of summer/beginning of autumn and in winter, under variable environmental conditions.

## 2. Materials and Methods

### 2.1. Animals and Rearing Conditions

Four Italian chicken breeds, Pepoi (PP), Ermellinata di Rovigo (ER), Robusta Maculata (RM) and Robusta Lionata (RL), were considered for the study. These breeds are classified as dual-purpose (meat and egg) breeds, even if differences in adult body size exist (Table 1). The body weight of adult females is 1.25 kg in PP, 2.30 kg in ER and 3.05 kg in RM and RL. Their origin is the Veneto region (Italy): PP is an old local breed; its origin is dated back to the XIX century, but no precise data on its genetic origin exist. The other breeds were created in Veneto during the 1950s: the ER breed originates from Sussex (tinted eggshell breed) and Rhode Island (brown eggshell breed) breeds, whereas for the RM and RL breeds, Brown Orpington and White America, two brown eggshell breeds were used [5]. In addition to the adult body weight, these breeds differ in their plumage colour: it is golden in PP, ER shows erminate plumage, RM plumage is predominantly white with black extremities in many regions of the body, black feathers of primaries and tail and RL shows a tawny plumage with black feathers of primaries and tail. All the breeds (Figure S1) show single comb and lay tinted eggshell eggs (Figure S2).

**Figure 1:**
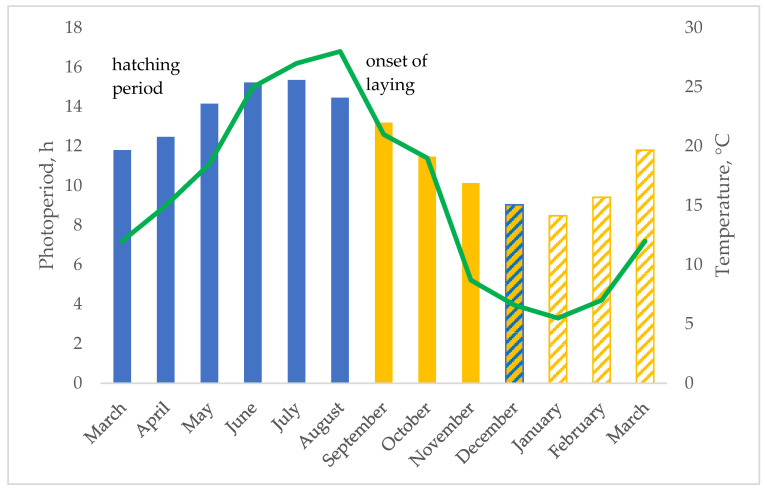
Photoperiod (bars) and outdoor temperature (line) from the hatching to the onset of laying and throughout the laying period. The bars indicate the length of the natural photoperiod throughout the life of the studied birds: growing period (full blue bars), first laying period (full yellow bars) under natural photoperiod, start of artificial lighting implementation (from the second half of month, diagonal blue-yellow bars), second laying period under implemented photoperiod (diagonal yellow bars).

Birds from each breed hatched at the same time (second half of March 2021) and were reared on the same farm of the Centre for Poultry Biodiversity Conservation in the Veneto region (it routinely provides for the rearing of these breeds as well as for the egg production for incubation and brooding), in Northern Italy (45°02′53″ N), from hatching to the pubertal age and throughout the laying period, which started from the end of summer to the beginning of autumn (Figure 1), according to the breed. Feeding, rearing conditions (photoperiod, temperature) and prophylaxis procedures were the same for the four breeds, from the time of hatching to the end of the studied period, which was in March 2022, at 53 weeks of age. The newly hatched chicks were kept indoors during the first 4 weeks of life, at an environmental temperature decreasing from 32 to 24 °C, under infra-red radiation lamps, on litter. At 1.5 months of age, the birds were given free access to outdoor spaces, from spring to autumn and winter. Each breed (60 hens) had free access to outdoor (5 m^2^/bird) space (equipped with linear drinkers), where the hens stayed throughout the day, and to indoor (4 hens/m^2^) space (wood shavings and straw litter, with perches and circular feeders), mainly used for laying eggs (collective nests) and at night; the area (indoor and outdoor) available to each breed was divided by netting.

At the start of laying (at about 22 weeks of age for the breed with earlier onset of laying), in September–October, depending on the breed, the photoperiod was natural and decreasing, according to the season and the geographical position of the trial station in Northern Italy (on average, 12L:12D), and for the second period (from 38 weeks of age, from December to 53 weeks of age), artificial light was gradually increased (inside the indoor space, incandescent bulb, about 60 lux) to obtain a photoperiod of 14L:10D. The photoperiod was regulated by a timer, and it was achieved by turning on the light one hour before sunrise and turning off it one hour after sunset. The external environmental temperature was 10 ± 3.4 °C and 11 ± 3.0 °C, respectively, for the first (from the beginning of October to the end of November) and the second (from the beginning of January to the end of March) laying periods, when the data on yield performance and egg quality were collected. The animals were given ad libitum commercial feeds according to their physiological phase (three diversified commercial feeds during the growing period). For the first laying phase (26–33 weeks of age, under decreasing natural photoperiod), when the breeds started laying, the birds were given a commercial pelleted feed (PG = 16%, ME = 11.8 MJ/kg, Ca = 1.1%, P = 0.6%) and maize (PG = 9.1%, ME = 13.7 MJ/kg), at a ratio of 1:1. It is worth remembering that, at this age, only the dual-purpose and tinted eggshell breeds showed a variable onset of laying and egg production rate, whereas the hens belonging to the other breeds of the Conservation Centre for Biodiversity and hatched at the same time of the studied breeds, such as the white eggshell breeds, did not start laying (Padovana breed) or they showed a very scant laying activity (Polverara breed), never reaching the threshold of 10% hen–day egg production. The reason for the choice of the Conservation Centre, to not use a feed for laying hens throughout this period, was due to not forcing the pullets to egg production throughout a season with decreasing temperature and photoperiod. The birds grew outdoors, under favourable conditions for skeletal health, walking on ground and under solar radiation. In the outdoor area for each breed, the presence of grass available to the birds was higher throughout the growing period than at the onset of laying, which occurred after the summer months, when the rain was scarce and did not allow for good grass regrowth. From 38 to 53 weeks of age, and under increasing photoperiod, the hens were fed a commercial pelleted feed for laying (PG = 16%, ME = 11.5 MJ/kg, Ca = 4.2%, P = 0.6%). All the feeds consisted mainly of maize and soybean.

### 2.2. Data Collection on Yield Performance and Egg Quality

The eggs were collected according to European Regulations (EC No. 1/2005 and EC No. 1099/2009) on animal care and welfare. The sampling did not affect the welfare of the hens as it was carried out when the animals were not in the nests, thus avoiding their handling. The egg production was checked daily, throughout two periods, from 26 to 33 weeks of age (autumn) and from 42 to 53 weeks of age (winter); the hen–day egg production was calculated as the number of eggs/number of live hens × 100.

At 32–33 and 52–53 weeks of age, samples of about 15–20 eggs (depending on the daily production of each breed), from a whole day’s production, were collected and weighed for each breed; the hen–day egg mass was calculated as hen–day egg production (%) x daily egg weight (g). At 32–33 and 53 weeks of age, external and internal egg traits were measured. For the first age, given the low laying rate showed by some breeds, the daily egg collection was carried out throughout two weeks to obtain representative egg samples and an adequate number of observations per each trait and breed. The number of eggs used for the physical analyses ranged, at first (32–33 weeks; the range, shown below, depends on the number of eggs laid, according to the laying activity of the breeds) and second (53 weeks) age, respectively, as follows: egg weight, diameters, eggshell colour 22–50 and 60; eggshell thickness 7–34 and 34; yolk, albumen and shell weight 7–17 and 25; yolk colour 7–14 and 8; Haugh units 7–16 and 22; blood and meat spots 13–64 and 60. The number of observations per each breed and trait is shown, in caption, under each table and figure. All the measurements were performed on a one-day-old egg.

The eggshell colour was measured using a colorimeter (Chroma meter CR 300, Minolta Co, Ltd., Osaka, Japan), using the CIE scale [22]: the L, a* and b* values reflect lightness (0 = black, 100 = white), redness (−100 = green, 100 = red) and yellowness (−100 = blue, 100 = yellow), respectively. The maximum length (longitudinal axis) and the maximum width (equatorial axis) of each egg were measured with callipers (0.01 mm), and the shape index was calculated as maximum width (MW)/maximum length (ML) × 100. As well as the egg shape and the position of the maximum width along the longitudinal axis are concerned, the minimum distance (MinDis) from the sharp end of the egg to the first point of MW was measured. Given that the MinDis from the half of ML (ML/2, MLh) may change, according to the egg shape (above the MLh or quite near), the difference between these two points was calculated (MLh − MinDis) and indicated as MWDif (maximum width difference). The ratio of MWDif on MLh was also calculated (MWDif/MLh × 100) and indicated as MWDifR (maximum width difference ratio).

Each egg was weighed, and the internal quality traits were measured. The eggshell was broken along the equatorial axis, the yolk was manually separated from the albumen, it was weighed and the albumen weight was calculated as the difference between the weight of the egg and the sum of the weight of the yolk and eggshell (after drying at 50 °C per 12 h). The ratio of each egg component (yolk, albumen and eggshell) was calculated as weight of each component/egg weight × 100. The total eggshell thickness (indicated as eggshell thickness) was measured using digital callipers (0.001 mm) (Mitutoyo, Japan). On samples of eggs at 53 weeks of age, the thickness of the layer of the internal eggshell membranes (carefully separated from the eggshell in the fresh egg, at the level of equatorial axis) and the thickness of the mineral layer were also measured using digital callipers (0.001 mm) (Mitutoyo, Japan). The proportion of each layer on the total eggshell thickness was calculated as the ratio of the thickness of each layer (membrane layer or mineral layer)/total eggshell thickness × 100. To calculate the Haugh Units (HU) [23], each egg was weighed and broken, and the yolk and albumen were put on a glass plate to measure the albumen height by means of a micrometre (0.01 mm) (Mitutoyo Co., Kawasaki, Japan). The yolk colour was measured using the DSM Yolk Fan (formerly Roche scale, 1–16, to distinguish the colour density). Blood and meat spots were visually evaluated on yolk and albumen, respectively, by means of a mirror positioned under the glass plate.

### 2.3. Statistical Analyses

Data on the hen–day egg production and hen–day egg mass, egg weight and the external (egg length, egg width, shape index, MWDif, MWDifR, eggshell colour traits, eggshell weight and eggshell ratio) and internal egg quality traits (weight and ratio of yolk, yolk colour, weight and ratio of albumen, yolk to albumen ratio, Haugh Units) were evaluated via ANOVA following a factorial model (4 × 2), considering breed and age as main effects and their interaction, and using the proc GLM of SAS (SAS, Institute, Cary, NC, USA). Given the different number of collected samples between the breeds and the two ages, the Type III SS (sum of squares) was considered. Per each age (26–33 weeks and 42–53 weeks), the effect of breed on the hen–day egg production was evaluated via a one-way ANOVA model, based on data for four (first laying age) and six (second laying age) periods (two weeks/period). Per each breed, to test linear, quadratic and cubic trends of the hen–day egg production, contrast statements were undertaken using orthogonal polynomial coefficients (SAS, Institute, Cary, NC, USA). For the data (at 53 weeks of age) on the ratio of each eggshell layer (mineral and membrane) on total thickness, as well as the total thickness and egg shape index, a one-way ANOVA model was used, considering breed as main effect. Significant differences among least square means were tested using Tukey’s test. Contrast estimates were performed for differences between ages, per each breed. Pearson’s correlations between the egg weight, albumen weight and yolk weight and the eggshell width and eggshell length were calculated, per each breed and age. Significant differences between breeds for the egg inclusions and between blood and meat spots per each breed were tested using χ^2^ test (SAS, Institute, Cary, NC, USA). Significance was set at *p* < 0.05.

## 3. Results

### 3.1. Yield Performance and Egg Weight

In Table 2, the hen–day egg production, hen–day egg mass and the egg weight of the four breeds are shown. The hen–day egg production throughout the entire studied period (from 26 to 33 weeks, and from 42 to 53 weeks of age) differed (*p* < 0.05) among the breeds, showing the highest percentage in ER, followed (*p* < 0.05) by PP, and the lowest in RL and RM (*p* < 0.05). The hen–day egg mass, referred to as 32–33 and 52–53 weeks of age, showed the highest (*p* < 0.05) value for ER, and RM was higher (*p* < 0.05) than PP and RL. The egg weight was higher (*p* < 0.05) in RL than in ER and RM, and PP was the lowest (*p* < 0.05).

**Figure 2:**
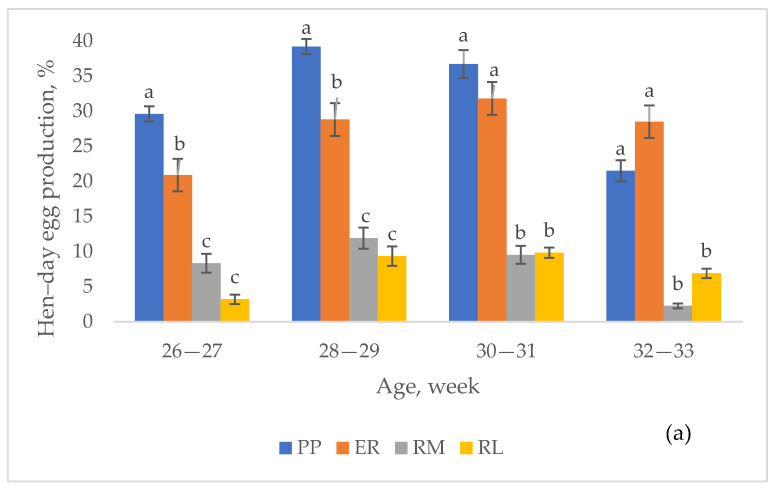

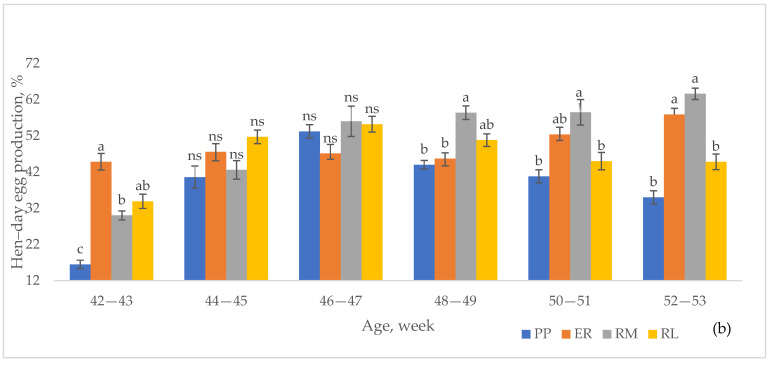
Effect of the breed on hen–day egg production (lsmeans ± SE) according to the age of the hens throughout first (**a**) and second (**b**) period. Different letters between breeds within age indicate different values. a, b, c: *p* < 0.05. ns: not significant. Breeds: Pepoi (PP), Ermellinata di Rovigo (ER), Robusta Maculata (RM), Robusta Lionata (RL). Observations (*n*): first period = 14 per each age and breed, second period = 14 per each age and breed.

In Figure 2, the effect of breed on the hen–day egg production, according to the age of the hens, and per each laying period, is shown. Throughout the first period (Figure 2a), PP was higher than ER (*p* < 0.05) from 26 to 29 weeks of age, and then they were similar; they were constantly higher (*p* < 0.05) than RM and RL, which were similar. At the second period (Figure 2b), each breed, with the exception of PP and RL, showed, at least once, a hen–day egg production higher (*p* < 0.05) than that of the other groups, according to the age. A higher hen–day egg production than the other groups was seen in ER at 42–43 (*p* < 0.05) and at 52–53 weeks (together to RM) in RM, from 48 to 53 weeks (*p* < 0.05). PP showed lower (*p* < 0.05) values than the other groups at 42–43 weeks, from 48 to 53 weeks, than RM and ER at 52–53 weeks. From 44 to 47 weeks, all the breeds showed the same hen–day egg production.

In Table 3, the trends of the hen–day egg production, according to the laying period, are shown per each breed. In the first period, from 26 to 33 weeks, the laying activity showed a quadratic trend for PP, RM and RL (*p* < 0.05) and a linear positive trend for ER (*p* < 0.05). In the second period, from 42 to 53 weeks, a linear positive trend was shown for ER and RM (*p* < 0.05) hens, whereas PP and RL birds showed a cubic trend (*p* < 0.05).

In Table 4, the estimates of the differences between the two ages for the yield performance and the egg weight are shown. All the breeds showed significant (*p* < 0.05) increases with ageing for all studied traits. The breeds showed widely different percentage increases in the hen–day egg production and hen–day egg mass: an extremely high increase was shown by RM, RL and, to a lesser extent, ER and PP. For these last two breeds, changes in hen–day egg production and egg weight, which affect the daily egg mass, showed a less marked increase with age.

**Figure 3:**
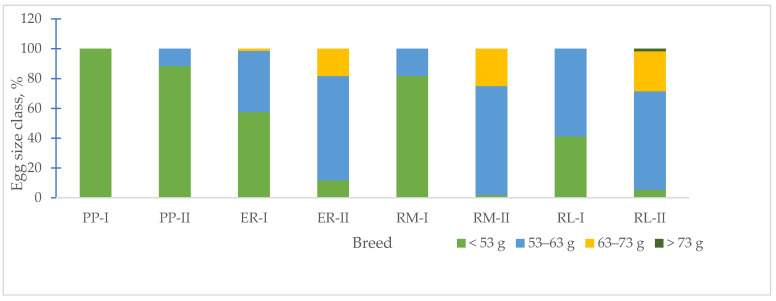
Effect of age on egg size class frequency per each breed. Breeds: Pepoi (PP), Ermellinata di Rovigo (ER), Robusta Maculata (RM), Robusta Lionata (RL). I = first laying period (26–33 weeks of age). II = second laying period (42–53 weeks of age). Observations (*n*): PP (40 at 32–33 weeks, 60 at 53 weeks), ER (36 at 32–33 weeks, 60 at 53 weeks), RM (22 at 32–33 weeks, 60 at 53 weeks), RL (51 at 32–33 weeks, 60 at 53 weeks).

According to the EC regulation [24] for egg weight, the size class distinguishes small (<53 g), medium (53–63 g), large (63–73 g) and very large (<73 g) egg sizes. In Figure 3, the changes in the frequency of the egg size class, according to the age of the hens, are shown. For the first age, the eggs of PP showed one size class, small (100%), RM and RL showed two size classes, small (RM = 81.8%, RL = 41.2%) and medium (RM = 18.2%, RL = 58.8%), and ER showed three size classes (small = 57.6%, medium = 40.9%, large = 1.5%). At the second age, when compared to the first age, ER, RM and RL showed a decreased percentage of small-size (ER = 11.7%, RM = 1.7%, RL = 5.4%) eggs and an increased percentage of medium- (especially, ER = 70.0% and RM = 73.3%, RL = 66.1%) and large-size (ER = 18.3%, RM = 25.0, RL = 26.8%) eggs. Only RL showed very large-size eggs (1.7%). PP showed two size classes, small and medium, with a lower percentage of medium-size (11.7 vs. 88.3%) eggs. Globally, the egg weight increased with ageing (Figure 3, Table 4) but to a different extent, according to the breed, as in PP, it reached 11.8%, in ER and RL 13.2%, and in RM 21.2%.

In this trial, the hen–day egg production differed among the breeds, reaching the highest value in ER, followed by PP, and the lowest in RM and RL. These breeds showed the same trends for the total number of eggs laid per hen, from 26 to 53 weeks of age, excluding the month of December (Figure S3). The data for each breed show a lower egg production when compared to the total number of eggs that a purebred hen annually produces, ranging from 150 (RM) to 180 (PP) [5], thus representing about 30% total egg production, even if the precise laying curve for each breed is unknown. The genetic origin of ER, RM and RL is known, as previously indicated, and their egg-laying rate reflects, almost partially, the productive attitude of the original breeds [6], whereas for the PP breed, as stated above, no indication exists on its origin. The increased hen–day egg production with the increasing age of hens, from 26–33 to 42–53 weeks of age, reflects, at least in part, the general oviposition curve of a laying hen, both for the effects of ageing of the birds and increasing photoperiod.

From the comparison with the laying curve of hybrid strains, the purebreds show differences, particularly lower egg production and persistence [8,25,26]. However, the observed trends, referring to the increasing age of the hens, need to be considered in the management of the month of hatching also. In fact, under outdoor rearing conditions, the physiological responses of the animals may vary, depending on the environmental conditions, such as temperature and photoperiod, which change according to the season and to the geographical position of the farm. In the current trial (Figure 1), the chicks hatched in spring, and the pubertal age was reached at the end of summer and the beginning of autumn, with differences according to the breed. The studied breeds differed for adult body weight and body conformation, as indicated in Table 1. The onset of laying depends on the growth rate and nutritional factors [7,27], but the interaction with changes in photoperiod and temperature may be a relevant factor. It is known that, in hybrids, the body weight of the pullet at 12–18 weeks is affected by the chick weight [28], but the season and the environmental temperature may also affect it. If during the last half of the growing period of a pullet, the light day decreases in length, the onset of egg production is delayed [29]. The nutritional requirements for body growth, and for the energy storage for body thermal regulation under the low temperatures of the cold winter season, as occurs in Northern Italy, for the young hens may be competitive factors with the nutrient requirements for egg formation [29]. As a consequence, the genetic asset of each breed may differently affect the physiological response of the young hen. The light perception, by the retina in the eye and through the cranial bones, may differ among the breeds for differences in the brain areas [29] also. In fact, only the studied breeds showed an onset of laying in autumn, whereas the other local breeds, reared on the same farm in the same period, and hatched at the same time as the studied breeds, such as Padovana (with cranial hernia) and Polverara (without cranial hernia), both tufted and white eggshell breeds, with adult body weight lower than those of ER, RM and RL [5], showed a more delayed onset of laying (Padovana) and a very scant egg production (Polverara) when compared to them and PP. It is worth remembering that Padovana and Polverara birds reached, at 26 weeks of age, 70–85% adult body weight, and only Polverara exceeded 80% (data provided by the Conservation Centre). In the current trial, the PP hens started laying earlier than ER, RM and RL. The PP hens showed an onset of laying more similar to that of ER, even if the ER onset was delayed by a few weeks. In the second period, after implementation of the natural photoperiod with artificial lighting, the egg production showed differences between the breeds in the first weeks and beyond.

The body growth rate and the percentage of adult body weight at the onset of laying are traits that characterize the hen genotype: these data exist for each hybrid strain [8,25,26], and differences among strains occur, especially when dual-purpose hybrids are compared to strains selected for high egg production, but the differences are small due to their genetic selection and improvement. Relevant differences among purebreds, not selected for any productive traits, and when compared to hybrid genotypes, were detected for the growth rate, body weight, egg production and onset of laying. The breeds of the current study are notably diversified (Table 1) for body size and muscle development. The RM and RL hens showed a more slowed down initial laying activity, especially RL, than ER and PP, as their marked dual-purpose attitude mainly related to meat production [5]. Previous indications on these breeds, referring to females at 24 weeks of age, reported significant (*p* < 0.01) differences between the live body weight of the three breeds and the same trend for the leg weight, with the highest value for RL and the lowest for ER; the breast weight was higher (*p* < 0.01) in RL and RM than in ER (Table 1) [21]. The comparison between the ER, RL and RM females showed increases in their body weight (11.6, 12.8, and 12.2%, respectively), breast muscle (5.0, 12.5, and 11.0%, respectively) and leg weight (4.7, 8.4 and 6.9%, respectively) from 20 to 24 weeks of age, with a superiority of the last two breeds, especially RL [21]. For the laying response of PP, in comparison to that of ER, it is possible that the adult body size of this breed is involved: for birds of a smaller size, complete body development and the final size, which could guarantee good egg formation along the oviduct, are generally reached earlier than those of hens belonging to breeds characterized by a higher adult body weight. In the present trial, at 26 weeks of age, PP hens reached adult body weight, whereas the other breeds (ER, RM, RL) had not completely reached it, as stated above (Table 1). For the chickens of local breeds reared outdoors, the onset of laying depends on the month of hatching: in Northern Italy, for birds born in spring and sexually mature between the end of summer and the beginning of autumn, the laying activity may start towards the last months of the year, even if under a decreasing photoperiod and environmental temperature. In autumn, at the latitude of Northern Italy, and under natural conditions, after the first weeks of laying activity, the laying rate slows down until reaching negligible values. It will start and increase again, at the beginning of spring in the new year, under an increasing natural photoperiod and environmental temperature. Producers may be interested in reaching high laying activity as soon as possible; thus, the implementation of a natural photoperiod with artificial lighting is used to increase the photoperiod. For the welfare of hens living outdoors, it would be a good practice to increase the photoperiod only at the beginning of the new year, when the birds should have reached a satisfactory development in terms of body size to guarantee good body thermal regulation and laying activity. In the current study, the hens showed different laying activity at the start of the second period. Particularly, RM and RL showed a constantly increasing egg production, whereas PP, after a light rise in the laying rate, showed a decreasing trend. The reasons for these responses may be due to a higher feed ingestion capacity and a more favourable body surface area to volume ratio, characterizing the RM and RL, than the PP breed and then a less conflicting utilization of the nutrients for body requirements and egg production. For such results, more knowledge is needed for an understanding of the effective body requirements of the hens according to the breeds and to the environmental seasonal conditions. In fact, the nutritional requirements are known for the hybrid strains [8,25,26], whereas precise literature indications do not exist for purebred hens [30]. When purebreds are compared, differences in the hen–day egg mass may be due not only to the egg-laying rate but also to the egg weight, especially when these two traits are notably different among the breeds. It is presumable that the hen–day egg mass of RM and RL were lower than those of ER and PP (hen–day egg mass of 2.7 g vs. 13.4 g, respectively, at 32–33 weeks of age), as the nutrient requests were still mainly related to their body development.

An important egg trait characterizing a breed is the egg weight (Figure 3). As known, the egg size changes with the age of the hen [15]; the changes in egg weight per each breed showed an increased frequency percentage of the medium- and large-size class for ER, RM and RL. For the PP eggs, the changes in size were smaller than those of the other groups. It seems that for this breed, characterized by a body weight of about 1.3 kg, reached at the beginning of the laying activity, from the onset of laying to 53 weeks of age, little changes occurred on the egg formation and size. Under outdoor rearing conditions, the egg weight could be affected by the delay in the onset of laying according to the natural environmental factors due to the season, but other factors, such as diet [31], size of the hens and breed, may be involved.

### 3.2. Eggshell Traits

In Table 5, the effects of breed on the eggshell traits are summarized.

The eggshell length was affected by breed, being higher (*p* < 0.05) in ER and RL than RM, and PP was the lowest (*p* < 0.05). The eggshell width was higher (*p* < 0.05) in RL than in ER, RM was intermediate and PP was the lowest (*p* < 0.05). The shape index was similar in ER and RL, which were lower (*p* < 0.05) than RM; PP was intermediate between RM and RL and higher (*p* < 0.05) than ER. The MWDif was higher (*p* < 0.05) in PP and RL than ER and RM; ER and RM were similar. The MWDifR was the highest (*p* < 0.05) in PP, followed by RL (*p* < 0.05), which was higher (*p* < 0.05) than ER and RM. The eggshell colour significantly (*p* < 0.05) differed among all the groups: the lightness was higher in PP than in RL, which was higher than ER, and RM was the lowest. On the contrary, the a* and b* index was higher (*p* < 0.05) in RM than in ER, and RL was higher (*p* < 0.05) than PP. RM showed higher (*p* < 0.05) eggshell thickness than RL, which showed higher (*p* < 0.05) values than those of PP, and ER was the lowest (*p* < 0.05).

In Table 6, the effect of age on the eggshell traits per each breed is shown. From 33 to 53 weeks of age, PP showed a significant increase (*p* < 0.05) in almost all the traits; only the lightness did not change with age. The ER eggs showed changes in the eggshell traits, showing positive (*p* < 0.05) values for the length, width, MWDif, lightness and thickness and negative (*p* < 0.05) for the egg shape index and a* index; the MWDifR and the b* index were not affected by age. The effect of age on the RM eggshell traits was significant (*p* < 0.05) and positive for the length, width, MWDif and thickness; the other traits were not affected by age. The RL eggshell showed an increase (*p* < 0.05) in length and width, MWDif, MWDifR and lightness and a decrease (*p* < 0.05) in the a* and b* index; the other traits did not change.

The effect of age was significant for almost all the eggshell traits for PP and ER and less for the other two breeds, especially RM. The breed affected the length and the width of the eggs; the effect on the shape of the eggshell is fixed by the shell membranes, as stated by Caswell Stoddard et al. [32], rather than by the shell itself. The body and the oviduct conformation, as well as the membrane material secretion, as affected by the genetic asset, are responsible for the final shape of the eggshell. At 53 weeks of age, the PP and RM eggshell showed significantly different and opposite proportions of the mineral and membrane layers (Figure S4a,b); although they showed a similar egg shape index, PP showed a higher MWDif and MWDifR (Table 6) and a significant increase in MWDif and MWDifR with age (Table 7). Given that the egg size of PP was smaller than that of RM, it is possible that in the PP eggs, the need for increasing dimensions involved an elongation of the membranes at the level of maximum width. Differently, the RM eggs, characterized by a higher weight and laid by hens with a higher body size than that of PP, seemed not to need such elongation at the level of the maximum width. The ER eggs showed the lowest egg shape index and the lowest total eggshell thickness, with a more balanced proportion of the membrane layer and mineral layer when compared to the other breeds (Figure S4a,b). More knowledge is needed for profiling the substructure of the eggshell of the studied breeds, as it may affect internal changes in the eggs during storage and brooding [12]. Other factors, such as the eggshell colour and pigment deposition, mainly set by genotype but also by diet and environmental conditions and their interactions, may affect eggshell traits [33]. Furthermore, these results indicate that for the PP eggs, showing, on average, a small size and lower length and width than those of the other breeds, the dimensions of the cartons for marketing should be considered. An egg external trait, which may identify the breed of the hen, is the eggshell colour. As dual-purpose breeds, the four breeds produce tinted eggshell eggs but with different pigment depositions, which affected the lightness and a* and b* indexes, which differed according to breed and, to a lesser extent, age. The colour may also change according to the age, as a dilution effect may occur [34]. The eggshell thickness, shape and weight are important traits, both for the producer and the consumer, and the variations throughout the laying curve may be relevant [35,36]. The eggshell thickness is related to the length of eggshell formation, and immature shell glands produce eggs with a thin eggshell [36]. The results of the current trial, for almost all the breeds, agree with the data previously shown by other authors [37] on the eggshell thickness from 20–24 weeks to 56–60 weeks of age. The effect of age on some eggshell traits may vary according to the environmental temperature and diet [7]. In the current trial, for the first period, the dietary calcium was lower than that suggested for hybrid pullets under an increasing photoperiod to stimulate sexual maturity [7]. The choice of such a diet was addressed to purebred birds reared outdoors under environmental conditions different from those of the intensive rearing system. It is worth remembering that, at the first age, for all breeds, the eggshell thickness values were into the range found by other authors [38]. Outdoor rearing, with walking on the ground and under the sun, is a very important condition for Ca metabolism and skeletal status for young females approaching laying activity [38].

### 3.3. Yolk and Albumen Traits, Egg Component Ratios and Correlations

In Table 7, the effect of breed on yolk and albumen traits and egg components ratio is shown.

The yolk weight differed among the genotypes, being higher (*p* < 0.05) in RM than in ER and RL, and PP was the lowest (*p* < 0.05). The albumen weight was higher in RL than in RM (*p* < 0.05), ER was intermediate and PP was the lowest (*p* < 0.05). The eggshell weight was higher (*p* < 0.05) in RM and RL than in PP and ER. RM showed a yolk ratio higher (*p* < 0.05) than that of PP and ER, and RL was the lowest (*p* < 0.05). ER and RL showed a higher (*p* < 0.05) albumen ratio than that of PP, which was higher (*p* < 0.05) than that of RM. The eggshell ratio was lower (*p* < 0.05) in ER than in the other groups. RM showed the highest (*p* < 0.05) yolk to albumen ratio, followed (*p* < 0.05) by PP and ER, which were higher (*p* < 0.05) than RL. The yolk colour did not change between the breeds. ER showed the highest (*p* < 0.05) HU, PP was lower (*p* < 0.05) than RM and RL was intermediate.

In Table 8, the effect of age on the changes in yolk and albumen traits and component ratios, according to each breed, is summarized. PP showed a significant increase in yolk, eggshell weight, eggshell ratio (*p* < 0.05) and albumen weight (*p* < 0.05), and a significant decrease (*p* < 0.05) in albumen ratio; the yolk ratio and the yolk to albumen ratio did not change. In ER, most of the traits significantly (*p* < 0.05) and positively changed, and the decrease (*p* < 0.05) was detected for the albumen ratio. The eggshell ratio did not change. RM and RL showed similar responses for the internal components. This showed a significant decrease in the eggshell ratio, whereas in RM, it did not change. Yolk colour and HU decreased (*p* < 0.05) with age in all the groups.

For the table eggs, the weight of the internal components, as well as their ratio, may be an important trait, especially when they differ from those of the hybrid eggs. The yolk to albumen ratio seems to be important for table eggs, as it may affect the nutritional value of the egg and the different use of eggs for cooking preparations [31]. With the ageing of hens, the yolk to albumen ratio increased only in ER, RM and RL. The effect of the breeder age on changes in internal components and eggshell, as stated before, is also important for supplying eggs with the best embryonic development, hatching performance and chick quality [12]. It is worth remembering that it seems that 51-week-old breeders of the slow-growing genotype produce heavier eggs and with an eggshell with better characteristics than those laid by 38-week-old hens [12]. More knowledge is needed for a better understanding of, for each breed, the most favourable time throughout the laying phase for obtaining eggs for the best embryonic development and hatching performance. The decreased colour score, shown by the eggs in this trial according to the age of the hens, may be due to the higher oviposition rate and yolk weight at the second age, which may have caused a dilution effect. Other factors, such as diet composition, metabolism and breed traits, such as the skin pigmentation, may affect deposition in body tissues of the ingested carotenoids [39,40].

Table 9 shows the correlations between the eggshell dimensions and the weight of the egg and those of its internal components per each breed, at 32–33 and 53 weeks of age. The egg weight showed a significant (*p* < 0.05) relationship with the eggshell width and eggshell length for PP, ER and RM; the RL egg weight was significantly (*p* < 0.05) correlated to the eggshell width at 32–33 and 53 weeks of age, and with the eggshell length, only at 53 weeks of age. The albumen weight was positively correlated (*p* < 0.05) to the eggshell width and length, at 32–33 weeks and 53 weeks of age, in PP and ER. Correlations between the albumen weight and the eggshell width were also significant (*p* < 0.05) for RM and RL, whereas the relationships with the eggshell length were not significant in RM and RL, at 32–33 weeks of age, and significant at 53 weeks of age both in RM and RL. A positive correlation exists (*p* < 0.05) between the yolk weight and the eggshell width and length in PP and ER, increasing with age for the width and slightly decreasing for the length. In RM, the yolk weight was positively (*p* < 0.05) correlated with the eggshell length, especially at first age, whereas no relationship was detected for the eggshell width. The RL yolk weight was correlated (*p* < 0.05) with the eggshell width, at both ages, whereas the correlation with the egg length was significant (*p* < 0.05) only at 53 weeks of age.

Stoddard et al. [32] stated that, broadly, in birds, the egg shape is correlated with the egg size and the hand-wing index. Furthermore, adaptations for flight influence the egg shape indirectly through the morphology of the pelvis, abdomen and oviduct. In the current trial, in the breed with the highest yolk weight, as well as the yolk to albumen ratio, as in RM, a higher correlation between yolk weight and eggshell length was observed with respect to the other groups. The ellipticity of an egg results because the membrane is easier to stretch along the oviduct axis then perpendicular to it. Asymmetry requires a difference in membrane material properties between the two poles [32]. In this study, the asymmetry was not calculated; only the changes in the minimum width along the longitudinal axis and the relationships between the egg components and dimensions vary according to the breed and to the age. These are the first data on the eggshell dimensions and the effect of yolk weight and the albumen synthesis on the membrane formation in the oviduct, and more studies are needed to know the mechanisms involving the eggshell formation and final egg shape of a breed.

**Figure 4:**
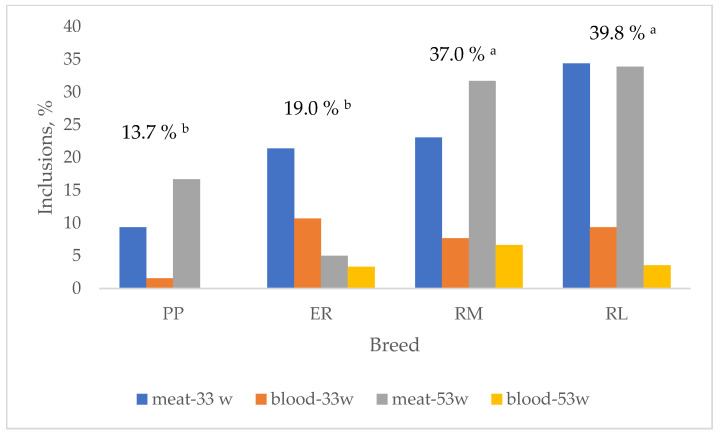
Effect of breed on total egg inclusions. Breeds: Pepoi (PP), Ermellinata di Rovigo (ER), Robusta Maculata (RM), Robusta Lionata (RL). The differently coloured bars indicate meat (albumen) and blood (yolk) inclusions per each age (-33 w = 32–33 weeks; -53 w = 53 weeks). Different letters between breeds indicate different values. a, b: *p* < 0.05; ns: not significant. Observations (*n*): PP (64 at 32–33 weeks, 60 at 53 weeks), ER (56 at 32–33 weeks, 60 at 53 weeks), RM (13 at 32–33 weeks, 60 at 53 weeks), RL (32 at 32–33 weeks, 56 at 53 weeks).

**Figure 5:**
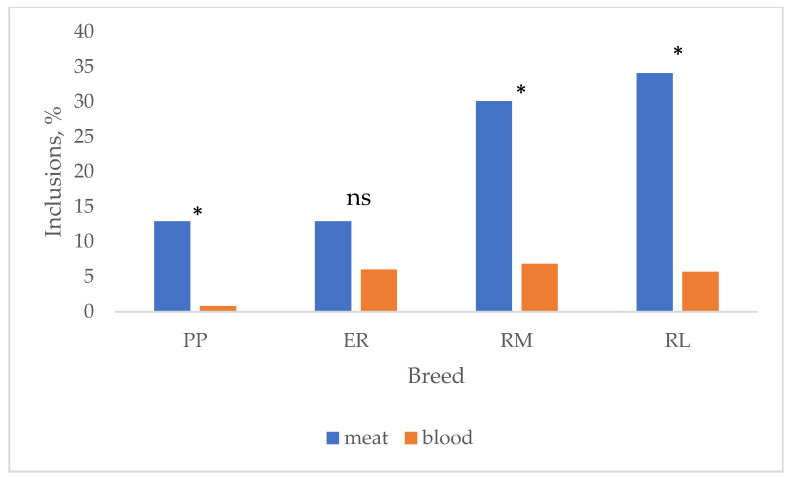
Effect of breed on blood and meat spots. Breeds: Pepoi (PP), Ermellinata di Rovigo (ER), Robusta Maculata (RM), Robusta Lionata (RL). * = different values (*p* < 0.05) between blood and meat spots. ns = not significant. Observations (*n*): PP = 124; ER = 116; RM = 73; RL = 88.

In Figure 4, the effect of breed on the total albumen and yolk inclusions and their changes according to the age are summarized. Total inclusions were lower (*p* < 0.05) in PP and ER than in RM and RL. These last two breeds showed quite homogeneous results for meat (RM: 33 w = 23.1%, 53 w = 31.7%; RL: 33 w = 34.4%, 53 w = 33.9%) and blood (RM: 33 w = 7.7%, 53 w = 6.7%; RL: 33 w = 9.4%, 53 w = 3.6%) spots according to the age, whereas PP (meat: 33 w = 9.4%, 53 w = 16.7%; blood: 33 w = 1.6%, 53 w = 0%) and ER (meat: 33 w = 21.4%, 53 w = 5.0%; blood: 33 w = 10.7%, 53 w = 3.3%) showed more diversified inclusion percentages. When total meat and blood inclusions are considered (Figure 5), PP (12.9 vs. 0.8%), RM (30.1 vs. 6.9%) and RL (34.1 vs. 5.7%) showed higher (*p* < 0.05) meat spots than blood spots, whereas ER (12.9 vs. 6.0%) showed only a tendential higher percentage of meat spots than blood spots. It is worth highlighting that the inclusion percentage showed by the studied breeds is high when compared to those exhibited by hybrid eggs [41] selected for this trait also. The effect of age on total inclusions varied according to the breed: throughout the first weeks of laying, the oviduct, especially the tracts involved with the albumen synthesis, is starting its activity, and more inclusions may occur, but also the implementation of the photoperiod may have affected the egg-laying rate and the oviduct activity.

## 4. Conclusions

The results add knowledge for profiling the egg production and quality of four Italian chicken breeds in the Veneto region. Their different genetic asset and interactions between physiological assets, body requirements and environmental conditions affected the productive responses of the hens, involving the onset of laying, the egg production and many egg traits. The overall results seem to indicate that the management of these breeds should be differentiated among them, especially between PP and ER, showing an earlier onset of laying and RM and RL. However, it seems that, also, between PP and ER, differences exist, and more studies are needed for their nutritional requirements under variable temperatures and photoperiods as well as for prospective selection schemes on productive and quality traits. Furthermore, further research should give knowledge for setting an optimal management of birds for each breed, in terms of month of hatching, to obtain good quality in their products and optimizing the welfare of the birds and the egg supplying, both for brooding and for human consumption, with the purpose of extended marketing for products throughout the year. For these dual-purpose breeds, meat production, from the males and from the hens, at the end of their productive cycle, is an important component for the overall economic evaluation of each breed.

## Figures and Tables

**Table 1:** Body traits of the hens belonging to the Italian dual-purpose breeds of the Veneto region.

	PP	ER	RM	RL
Skin colour ^1^	yellow	yellow	yellow	yellow
Wingspan ^2^, cm	37.2	46.0	46.2	46.7
Adult body weight ^1,2^, kg	1.25	2.30	3.05	3.05
Adult body weight at 26 weeks ^1^, %	100	90	84	88
Breast at 24 weeks ^3^, g	-	291	383	396
Leg at 24 weeks ^3^, g	-	449	499	544

Breeds: Pepoi (PP), Ermellinata di Rovigo (ER), Robusta Maculata (RM), Robusta Lionata (RL). ^1^ [5]. ^2^ [20]. ^3^ [21].

**Table 2:** Effect of the breed on yield performance and egg weight throughout the two laying periods.

	PP	ER	RM	RL	*p*	RMSE
Hen–day egg production ^1^, %	34.9 ^b^	38.3 ^a^	29.6 ^c^	27.0 ^c^	<0.0001	10.49
Hen–day egg mass ^2^, g	15.0 ^c^	23.7 ^a^	18.7 ^b^	15.4 ^c^	<0.0001	4.67
Egg weight ^3^, g	45.9 ^c^	55.3 ^b^	55.3 ^b^	57.1 ^a^	<0.0001	4.02

Different letters within traits indicate different values a, b, c: *p* < 0.05. RMSE = Root Mean Square Error. Breeds: Pepoi (PP), Ermellinata di Rovigo (ER), Robusta Maculata (RM), Robusta Lionata (RL). ^1^ Observations (*n*): 141 per each breed. ^2^ Observations (*n*): 28 per each breed. ^3^ Observations (*n*): PP (100), ER (96), RM (82), RL (107).

**Table 3:** Orthogonal contrasts for linear, quadratic and cubic component on hen–day egg production of the breeds according to the age of the hens.

	PP	ER	RM	RL
From 26 to 33 weeks	quadratic *	linear *	quadratic *	quadratic *
From 42 to 53 weeks	cubic *	linear *	linear *	cubic *

*: *p* < 0.05. Breeds: Pepoi (PP), Ermellinata di Rovigo (ER), Robusta Maculata (RM), Robusta Lionata (RL). Observations (*n*): from 26 to 33 weeks = 56 per breed, from 42 to 53 weeks = 85 per breed.

**Table 4:** Effect of age (contrast estimates ± SE, and percent variation) on yield performance and egg weight.

	PP	%	ER	%	RM	%	RL	%
Hen–day egg production, %	6.36 ± 2.02 *	20.2	21.7 ± 1.49 *	78.9	43.3 ± 2.14 *	541	39.4 ± 1.45 *	537
Hen–day egg mass, g	8.30 ± 1.46 *	76.1	15.9 ± 1.94 *	101	34.7 ± 2.10 *	242	22.9 ± 1.47 *	575
Egg weight ^1^, g	5.11 ± 0.67 *	11.8	6.90 ± 0.95 *	13.3	10.6 ± 0.89 *	21.2	7.01 ± 0.88 *	13.1

*: *p* < 0.05. Breeds: Pepoi (PP), Ermellinata di Rovigo (ER), Robusta Maculata (RM), Robusta Lionata (RL). Observations (*n*): from 26 to 33 weeks = 56 per breed, from 42 to 53 weeks = 85 per breed. ^1^ Observations (*n*): PP (40 at 32–33 weeks, 60 at 53 weeks), ER (36 at 32–33 weeks, 60 at 53 weeks), RM (22 at 32–33 weeks, 60 at 53 weeks), RL (51 at 32–33 weeks, 60 at 53 weeks).

**Table 5:** Effect of the breed on eggshell traits.

	PP	ER	RM	RL	*p*	RMSE
Eggshell length, cm	5.22 ^c^	5.63 ^a^	5.52 ^b^	5.66 ^a^	<0.0001	0.21
Eggshell width, cm	3.95 ^c^	4.19 ^b^	4.21 ^ab^	4.24 ^a^	<0.0001	0.12
Shape index	75.6 ^ab^	74.4 ^c^	76.5 ^a^	75.0 ^bc^	<0.0001	2.97
MWDif ^1^, cm	0.258 ^a^	0.157 ^b^	0.167 ^b^	0.227 ^a^	<0.0001	0.11
MWDifR ^2^, %	9.88 ^a^	5.60 ^c^	6.07 ^c^	8.03 ^b^	<0.0001	3.94
L	84.9 ^a^	73.8 ^c^	68.0 ^d^	77.6 ^b^	<0.0001	4.10
a*	2.17 ^d^	9.28 ^b^	11.1 ^a^	6.42 ^c^	<0.0001	2.47
b*	13.8 ^d^	21.7 ^b^	23.9 ^a^	19.0 ^c^	<0.0001	3.46
Eggshell thickness ^3^, μm	329 ^c^	311 ^d^	360 ^a^	343 ^b^	<0.0001	25

Different letters within traits indicate different values. a, b, c, d: *p* < 0.05. RMSE = Root Mean Square Error. Breeds: Pepoi (PP), Ermellinata di Rovigo (ER), Robusta Maculata (RM), Robusta Lionata (RL). ^1^ MWDif = maximum length/2 − MinDis. MinDis = distance from the sharp end of the egg to the first point of maximum width. ^2^ MWDifR = MWDif/(maximum length/2) × 100. Observations (*n*): PP (100), ER (96), RM (80), RL (100). ^3^ Observations (*n*): PP (69), ER (63), RM (41), RL (48).

**Table 6:** Effect of age (contrast estimates ± SE and percent variation) on eggshell traits.

	PP	%	ER	%	RM	%	RL	%
Length, cm	0.114 ± 0.042 *	2.1	0.345 ± 0.042 *	6.4	0.361 ± 0.060 *	6.7	0.240 ± 0.041 *	4.3
Width, cm	0.186 ± 0.021 *	4.9	0.125 ± 0.029 *	3.2	0.265 ± 0.025 *	6.6	0.174 ± 0.024 *	4.3
Egg shape index	1.99 ± 0.592 *	2.7	−2.36 ± 0.606 *	−3.2	−0.188 ± 0.843 ns	−0.3	−0.122 ± 0.541 ns	−0.3
MWDif ^1^, cm	0.109 ± 0.020 *	53	0.051 ± 0.023 *	40	0.071 ± 0.031 *	54	0.074 ± 0.020 *	39
MWDifR ^2^, %	3.98 ± 0.782 *	51	1.46 ± 0.806 ns	30	2.09 ± 1.113 ns	41	2.29 ± 0.716 *	39
L	−0.110 ± 0.654 ns	−0.1	6.61 ± 0.905 *	9.4	−0.738 ± 0.852 ns	−1.2	6.48 ± 1.01 *	8.8
a*	1.27 ± 0.371 *	83	−2.61 ± 0.555 *	−25	1.10 ± 0.615 ns	11	−3.47 ± 0.573 *	−43
b*	1.73 ± 0.728 *	14	−1.21 ± 0.710 ns	−5.4	0.621 ± 0.622 ns	2.5	−3.77 ± 0.803 *	−18
Thickness ^3^, μm	21.5 ± 5.75 *	6.9	19.7 ± 7.12 *	6.6	28.6 ± 9.58 *	8.4	−12.9 ± 7.54 ns	−3.7

*: *p* < 0.05. ns: not significant. Breeds: Pepoi (PP), Ermellinata di Rovigo (ER), Robusta Maculata (RM), Robusta Lionata (RL). ^1^ MWDif = maximum lenght/2 − MinDis. MinDis = distance from the sharp end of the egg to the first point of maximum width. ^2^ MWDifR = MWDif/(maximum length/2) × 100. Observations (*n*): PP (40 at 32–33 weeks, 60 at 53 weeks), ER (36 at 32–33 weeks, 60 at 53 weeks), RM (22 at 32–33 weeks, 60 at 53 weeks), RL (51 at 32–33 weeks, 60 at 53 weeks). ^3^ Observations (*n*): PP (34 at 32–33 weeks, 35 at 53 weeks), ER (28 at 32–33 weeks, 35 at 53 weeks), RM (7 at 32–33 weeks, 34 at 53 weeks), RL (16 at 32–33 weeks, 32 at 53 weeks).

**Table 7:** Effect of breed on the yolk and albumen traits and egg component ratio.

	PP	ER	RM	RL	*p*	RMSE
Weight, g						
Yolk	13.7 ^c^	16.3 ^b^	17.6 ^a^	16.0 ^b^	<0.0001	1.44
Albumen	27.8 ^c^	33.8 ^ab^	33.2 ^b^	35.1 ^a^	<0.0001	2.60
Eggshell	4.47 ^b^	4.64 ^b^	5.30 ^a^	5.34 ^a^	<0.0001	0.48
Ratio, %						
Yolk	29.7 ^b^	29.7 ^b^	31.2 ^a^	28.3 ^c^	<0.0001	1.73
Albumen	60.6 ^b^	61.8 ^a^	59.3 ^c^	62.2 ^a^	<0.0001	1.71
Eggshell	9.72 ^a^	8.47 ^b^	9.45 ^a^	9.49 ^a^	<0.0001	0.62
Yolk/albumen	0.492 ^b^	0.481 ^b^	0.528 ^a^	0.457 ^c^	<0.0001	0.04
Yolk colour ^1^	7.96	8.17	8.26	8.83	<0.0001	1.03
HU ^2^	105 ^b^	109 ^a^	102 ^c^	104 ^bc^	<0.0001	3.94

Different letters within traits indicate different values. a, b, c: *p* < 0.05. RMSE = Root Mean Square Error. Breeds: Pepoi (PP), Ermellinata di Rovigo (ER), Robusta Maculata (RM), Robusta Lionata (RL). Observations (*n*): PP (42), ER (42), RM (32), RL (40). ^1^ Observations (*n*): PP (23), ER (20), RM (16), RL (16). ^2^ Observations (*n*): PP (38), ER (38), RM (28), RL (29).

**Table 8:** Effect of age (contrast estimates ± SE and percent variation) on yolk and albumen traits and egg component ratio.

	PP	%	ER	%	RM	%	RL	%
Weight, g								
-Yolk	1.63 ± 0.402 *	13	3.02 ± 0.461 *	20	3.55 ± 0.683 *	23	2.84 ± 0.472 *	19
-Albumen	1.78 ± 0.714 *	6.7	2.57 ± 0.756 *	8.0	3.46 ± 1.13 *	11	2.97 ± 0.992 *	8.9
-Eggshell	0.661 ± 0.140 *	16	0.467 ± 0.181 *	11	0.927 ± 0.201 *	19	0.143 ± 0.138 ns	2.9
Ratio, %								
-Yolk	0.942 ± 0.587 ns	3.4	2.23 ± 0.496 *	7.7	2.02 ± 0.775 *	6.6	2.15 ± 0.539 *	8.1
-Albumen	−1.52 ± 0.559 *	−2.5	−2.15 ± 0.494 *	−3.3	−2.35 ± 0.723 *	−4.0	−1.40 ± 0.583 *	−2.2
-Eggshell	0.582 ± 0.213 *	6.0	−0.076 ± 0.184 ns	−0.8	0.277 ± 0.263 ns	3.0	−0.753 ± 0.202 *	−7.6
Yolk to albumen	0.027 ± 0.013 ns	5.7	0.053 ± 0.012 *	12	0.055 ± 0.020 *	11	0.045 ± 0.013 *	10
Yolk colour ^1^	−1.93 ± 0.406 *	−22	−1.67 ± 0.470 *	−19	−3.19 ± 0.401 *	−32	−3.67 ± 0.661 *	−35
HU ^2^	−15.6 ± 1.27 *	−13	−16.9 ± 1.30 *	−15	−15.8 ± 1.69 *	−15	−19.0 ± 1.64 *	−17

*: *p* < 0.05; ns: not significant. Breeds: Pepoi (PP), Ermellinata di Rovigo (ER), Robusta Maculata (RM), Robusta Lionata (RL). Observations (*n*): PP (16 at 32–33 weeks, 26 at 53 weeks), ER (17 at 32–33 weeks, 25 at 53 weeks), RM (7 at 32–33 weeks, 25 at 53 weeks), RL (16 at 32–33 weeks, 24 at 53 weeks). ^1^ Observations (*n*): PP (14 at 32–33 weeks, 9 at 53 weeks), ER (11 at 32–33 weeks, 9 at 53 weeks), RM (7 at 32–33 weeks, 9 at 53 weeks), RL (9 at 32–33 weeks, 7 at 53 weeks). ^2^ Observations (*n*): PP (16 at 32–33 weeks, 22 at 53 weeks), ER (16 at 32–33 weeks, 22 at 53 weeks), RM (7 at 32–33 weeks, 21 at 53 weeks), RL (9 at 32–33 weeks, 20 at 53 weeks).

**Table 9:** Pearson’s correlations between eggshell traits and egg, yolk and albumen weight of eggs laid by the breeds at 32–33 and 53 weeks of age.

	32–33 Weeks ^1^		53 Weeks ^2^	
	Eggshell Width	Eggshell Length	Eggshell Width	Eggshell Length
PP				
Egg weight	0.80 *	0.81 *	0.94 *	0.76 *
Albumen weight	0.70 *	0.77 *	0.84 *	0.81 *
Yolk weight	0.62 *	0.50 *	0.85 *	0.44 *
ER				
Egg weight	0.85 *	0.81 *	0.86 *	0.71 *
Albumen weight	0.84 *	0.76 *	0.73 *	0.67 *
Yolk weight	0.68 *	0.78 *	0.74 *	0.50 *
RM				
Egg weight	0.94 *	0.90 *	0.73 *	0.62 *
Albumen weight	0.97 *	0.76 ns	0.77 *	0.42 *
Yolk weight	0.78 ns	0.99 *	0.26 ns	0.75 *
RL				
Egg weight	0.87 *	0.24 ns	0.93 *	0.68 *
Albumen weight	0.77 *	0.31 ns	0.88 *	0.68 *
Yolk weight	0.82 *	0.14 ns	0.74 *	0.45 *

*: *p* < 0.05; ns: not significant. Breeds: Pepoi (PP), Ermellinata di Rovigo (ER), Robusta Maculata (RM), Robusta Lionata (RL). ^1,2^ Observations (*n*): PP (16 at 32–33 weeks, 25 at 53 weeks), ER (17 at 32–33 weeks, 25 at 53 weeks), RM (5 at 32–33 weeks, 25 at 53 weeks), RL (16 at 32–33 weeks, 25 at 53 weeks).

## Data Availability

The data presented in this study are available on reasonable request from the corresponding author. The data are not publicly available due to their sensitive nature.

## Supplementary Materials

The following supporting information can be downloaded at: https://www.mdpi.com/article/10.3390/ani13193064/s1, Figure S1: Phenotypes (male and female) of Pepoi (a), Ermellinata di Rovigo (b), Robusta Maculata (c) and Robusta Lionata (d). Figure S2: Eggs of Pepoi, Ermellinata di Rovigo, Robusta Maculata and Robusta Lionata. Figure S3: Number of eggs laid per hen, from 26 to 33 weeks of age and from 42 to 53 weeks of age, and eggs laid per hen as % annual laying phase, for the studied breeds. Figure S4: Effect of breed on mineral layer and eggshell thickness (a) and on membrane layer and egg shape index (b), at 53 weeks of age.
