# Supplementary material for: A Study on Egg Production and Quality According to the Age of Four Italian Chicken Dual-Purpose Purebred Hens Reared Outdoors
Source: Animals (Basel). 2023 Sep 29;13(19):3064. doi: 10.3390/ani13193064 (PMC10571830; doi:10.3390/ani13193064)
Supplement: Supplementary file 1 [file animals-13-03064-s001.zip › animals-2536663-supplementary.pdf]

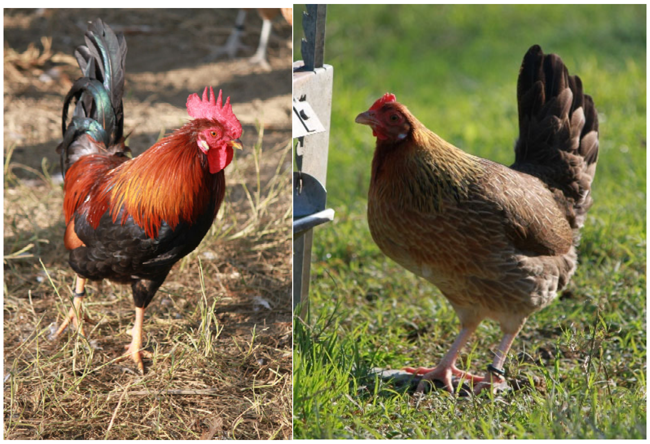
(a)

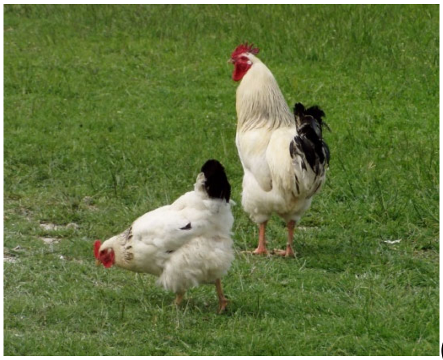
(b)

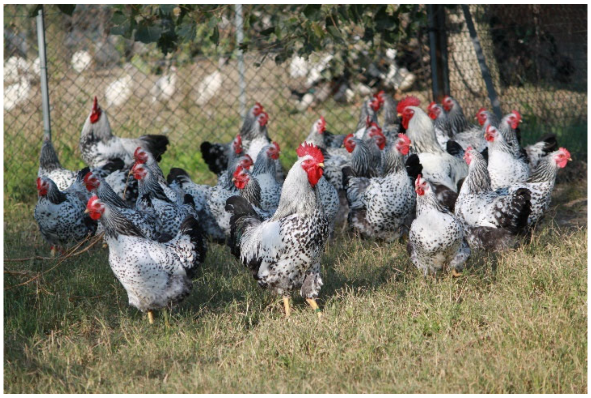
(c)

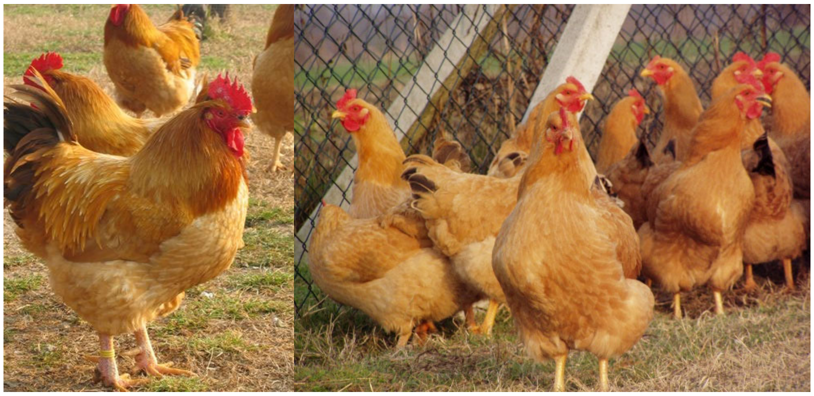
(d)

Figure S1. Phenotypes (male and female) of Pepoi (a), Ermellinata di Rovigo (b), Robusta maculata (c) and Robusta lionata (d).

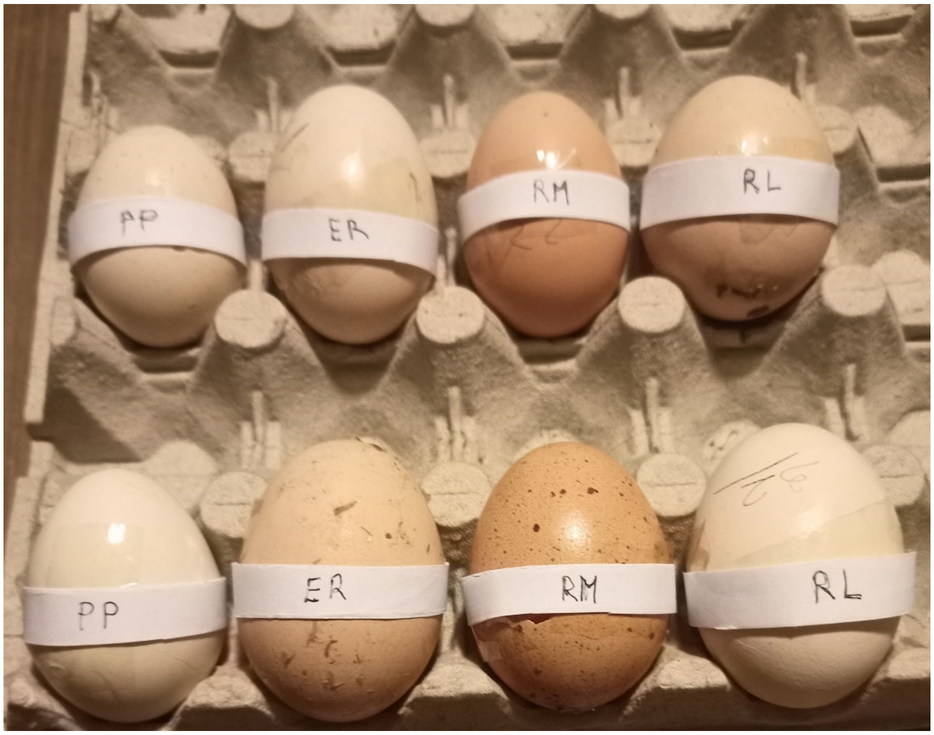

Figure S2. Eggs of Pepoi (PP), Ermellinata di Rovigo (ER), Robusta maculata (RM) and Robusta lionata (RL).

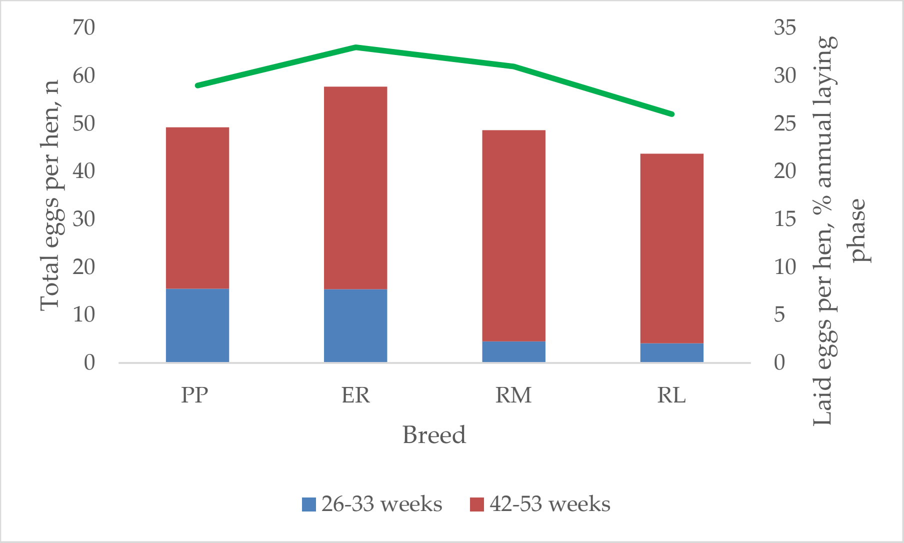

Figure S3: Number of eggs laid per hen, from 26 until to 33 weeks of age (blue column) and from 42 until to 53 weeks of age (red column), and eggs laid per hen as % annual laying phase (green line), for the studied breeds. Breeds: Pepoi (PP), Ermellinata di Rovigo (ER), Robusta maculata (RM), Robusta lionata (RL).

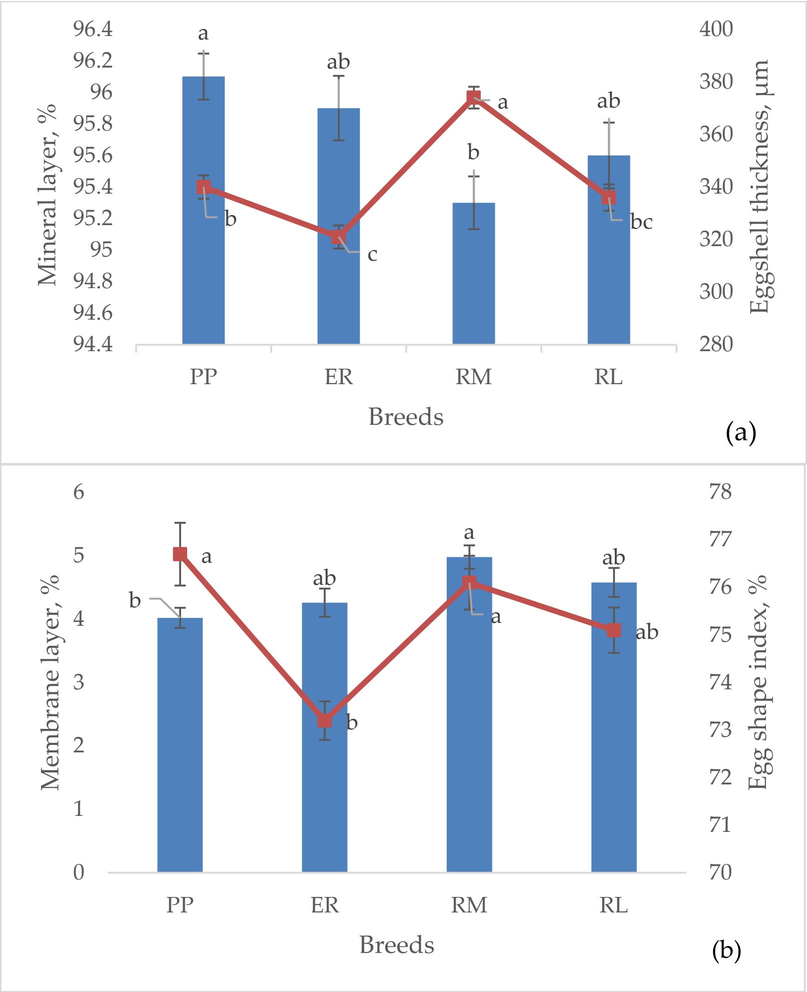

Figure S4. Effect of breed on mineral layer (lsmeans ± SE) (bars) and eggshell thickness (lsmeans ± SE) (line) (a) and on membrane layer (lsmeans ± SE) (bars) and egg shape index (lsmeans ± SE) (line) (b), at 53 weeks of age. Different letters between breeds within traits (bars and line pointers) indicate different values. a, b: $p < 0.05$. Breeds: Pepoi (PP), Ermellinata di Rovigo (ER), Robusta maculata (RM), Robusta lionata (RL). Observations (*n*): PP = 35, ER = 35, RM = 34, RL = 32.

In Figure S4, the effect of breed on the proportions of the mineral layer (Figure 4a) and of the membrane layer (Figure 4b), at 53 weeks of age, is shown, together to the eggshell thickness (Figure 4a) and the egg shape index (Figure 4b), respectively. The PP eggs showed the highest ($p < 0.05$) proportion of the mineral layer and the lowest ($p < 0.05$) proportion of the membrane layer, whereas the RM eggs showed opposite results, the lowest ($p < 0.05$) proportion of the mineral layer and the highest ($p < 0.05$) proportion of the membrane layer. The ER and RL eggshell showed intermediate results. For the eggshell thickness (Figure 4a), PP and RM showed ($p < 0.05$) opposite trends and differences than those of the mineral proportion; ER was lower ($p < 0.05$) than RM and PP. For the egg shape index (Figure 4b), PP and RM were similar and higher ($p < 0.05$) than ER, and RL was intermediate.
